# A Systematic Review and Meta-Analysis on Possible Role of Vitamin C in Sepsis

**DOI:** 10.7759/cureus.32886

**Published:** 2022-12-23

**Authors:** Jonathan Brown, Cassie Robertson, Luis Sevilla, Jorge Garza, Hytham Rashid, Ana C Benitez, Mikhail Shipotko, Zuhair Ali

**Affiliations:** 1 Internal Medicine, University of Houston College of Medicine; HCA Houston Healthcare Kingwood, Kingwood, USA; 2 Internal Medicine, Corpus Christi Medical Center, Corpus Christi, USA; 3 Graduate Medical Education, HCA Physician Services Group, Gulf Coast Division, Houston, USA

**Keywords:** mortality, septic shock, sepsis, ascorbic acid, vitamin c

## Abstract

Sepsis is a substantial healthcare burden, and its management continues to be a major challenge. Prior studies demonstrate conflicting evidence regarding the utility of vitamin C in sepsis. This systematic review and meta-analysis aim to collect data among critically ill patients (sepsis/septic shock), comparing the efficacy of parenteral vitamin C with standard care.

A literature review was conducted using databases including PubMed, Web of Science, Google Scholar, and the Cochrane Library to identify randomized controlled trials (RCTs) and observational studies comparing intravenous vitamin C alone or in combination with thiamine or glucocorticoids to the standard of care. We identified 11 RCTs and seven retrospective cohort studies. The primary outcome was 28-day mortality. Secondary outcomes included intensive care unit (ICU) length of stay, change in Sequential Organ Failure Assessment (SOFA) score, duration of vasopressor use, and duration of mechanical ventilation.

A total of 18 studies with 4078 patients were included in our final analysis. Overall, we found no mortality reduction in patients treated with vitamin C compared to standard of care (odds ratio (OR) 0.92; 95% confidence interval (CI) 0.78 to 1.09; p = 0.34). Studies that reported a change in SOFA scores, ICU length of stay, duration of mechanical ventilation, or duration of vasopressor use did not show any significant difference between groups. Subgroup analysis with RCT versus observational studies and vitamin C dosage regimens did not show any difference.

Among patients with sepsis or septic shock, treatment with vitamin C was not associated with a reduction in mortality, ICU length of stay, change in SOFA score, duration of vasopressor use, or duration of mechanical ventilation. Further studies are needed to demonstrate the potential role of vitamin C in the management of sepsis.

## Introduction and background

Sepsis is one of the leading causes of death and patients suffering from severe sepsis often require intensive care treatment and account for a large portion of intensive care unit (ICU) resource consumption [1]. A multimodal approach to treatment is essential. Vitamin C has been hypothesized to be a cost-effective adjuvant therapy with the ability to prevent multiorgan failure and improve mortality [2].

Vitamin C, or ascorbic acid, is a water-soluble vitamin, antioxidant, and essential cofactor for several enzymes including dopamine β-hydroxylase and peptidylglycine α-amidating monooxygenase which synthesize norepinephrine and vasopressin [3,4]. Animal models demonstrated the ability of vitamin C to preserve microvascular function including arteriolar responsiveness to vasoconstrictors [5]. In addition, vitamin C has been shown to exhibit bacteriostatic activity against mycobacteria and enhance the effect of broad-spectrum antibiotics via a synergistic effect [6,7].

With the inability to endogenously synthesize vitamin C, humans are dependent upon exogenous intake of vitamin C for anti-inflammatory and immune regulatory function [8]. Due to increased metabolism, critically ill patients with septic shock have hypovitaminosis C despite receiving standard parenteral nutrition [9]. Rapid correction of hypovitaminosis C through intravenous administration has been found to decrease the risk of organ failure and lower pro-inflammatory markers in septic patients [10]. Prior studies showed that supraphysiologic doses of vitamin C are required for correction among septic and critically ill patients [9,11]. The primary focus of this review is to determine whether vitamin C has a role in decreasing mortality in critically ill patients with sepsis.

## Review

Methodology

Study Design and Study Selection

**Figure 1:**
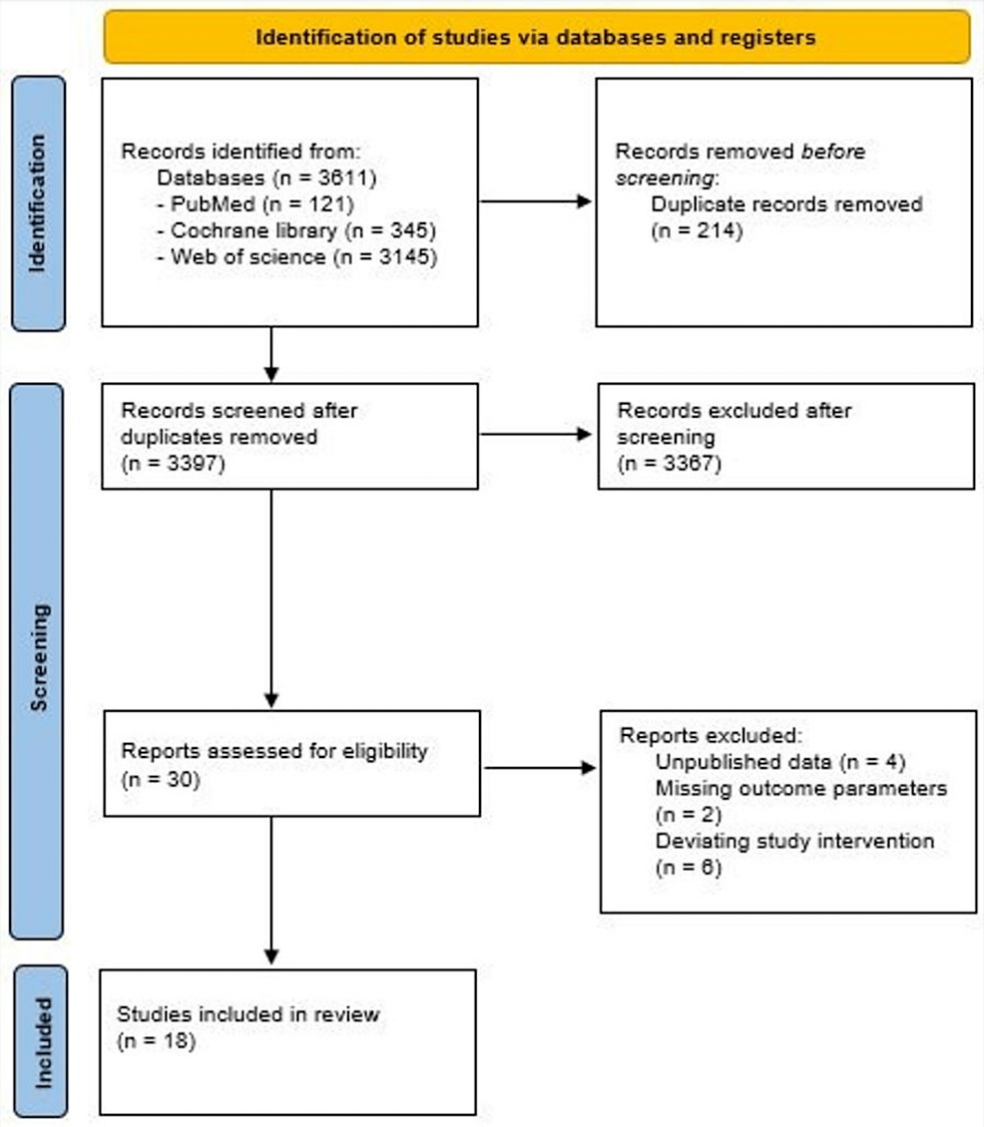
PRISMA 2020 Flow Diagram. PRISMA: Preferred Reporting Items for Systematic Reviews and Meta-Analysis

A systematic review was conducted according to the guidelines of the Preferred Reporting Items for Systematic Reviews and Meta-Analysis (PRISMA) statement. The protocol was registered in the PROSPERO international prospective register of systematic review (registration number CRD42021286473) on November 20, 2021. A literature search was conducted to review all articles from January 1, 2000, to December 31, 2021. The search included randomized controlled trials (RCTs), and prospective and retrospective observational studies enrolling patients with sepsis or septic shock. Search terms included “vitamin C,” “ascorbic acid,” “sepsis,” “severe sepsis,” “treatment,” and “septic shock.” Two independent investigators (L.S. and B.C.) searched PubMed, Web of Science, Google Scholar, and the Cochrane Library to identify articles for a full review. Any discrepancies were discussed and resolved by the principal investigator (Z.A.). Additional literature was discovered using the Google Scholar database with the above search terms and by using the reference section of articles found through the PubMed search. Search results were further limited to studies written in the English language. Preference for selection was given to clinical trials that assessed the utility of vitamin C administration to septic patients. Eighteen articles met the criteria for use in this review (Figure 1).

Eligibility criteria

We included observational studies and RCTs, blinded or unblinded, and single or multicenter. The patient population included adult patients ≥ 18 years of age with one or more of the following diagnoses: sepsis, severe sepsis, or septic shock (as defined by authors of individual studies). The intervention included intravenous vitamin C alone or in combination with thiamine and/or glucocorticoids compared to a matching placebo. The primary outcome of interest was 28-day mortality. Studies were assessed for the ability to provide data on mortality rates and timing. Secondary endpoints included mean difference (MD) in ICU length of stay (LOS) (in days), Delta Sequential Organ Failure Analysis (SOFA) score, duration of vasopressor use, and duration of mechanical ventilation.

Statistical analysis

The effects of the intervention on 28-day mortality were investigated by assessing the OR between the vitamin C and control group by pooling the available data. OR and pooled OR were determined and presented using the Forest plots with 95% confidence intervals (CI). A fixed or random effects model (Mantel-Haenszel) was used to pool data where appropriate. Statistical heterogeneity between the studies was evaluated using Cochran’s Q test and I2 statistics. Publication bias for the outcomes was explored visually with funnel plots. Asymmetry in the funnel plot was considered as a presence of publication bias which was tested statistically with the Harbord test.

Subgroup analysis was performed for secondary outcomes by using a two-sample t-test to assess the MD between the two groups. Furthermore, when appropriate, time measured in hours was converted to days. Values were expressed as mean ± standard deviation (SD). The statistical analysis was performed using STATA version 17 (StataCorp. 2021. Stata Statistical Software: Release 17. College Station, TX: StataCorp LLC.). A two-sided p-value ≤ 0.05 was considered statistically significant.

Results

A total of 18 observational studies and RCTs consisting of 4078 patients were included in the final analysis. Twelve studies were excluded due to either unpublished data, missing outcome parameters, or deviating study interventions. RCTs included patients with sepsis or septic shock, burn patients, and surgically critical sepsis patients admitted to the ICU. The characteristics of the included studies are summarized in Table 1.

**Table 1:** Study summaries. ED: emergency department; ICD: international classification of diseases; ICU: intensive care unit; LOS: length of stay; RCT: randomized controlled trial; RRT: renal replacement therapy; SOFA: sequential organ failure assessment; TBSA: total body surface area

Author, Year Published, Country	Study Design/Period	Sample Size	Population	Intervention	Outcome
Sevransky et al., 2021, USA [12]	Multicenter RCT (August 2018 to July 2019)	501	Patients with sepsis-induced respiratory and/or cardiovascular dysfunction	IV vitamin C (1.5 g), thiamine (100 mg), hydrocortisone (50 mg) q6 hr; n =252. Matching placebo for 96 hr or until discharged from ICU or death; n = 249	Primary outcome: number of consecutive ventilator and vasopressor-free days in the first 30 days following randomization. Secondary outcome: 30-day mortality
Fujii et al., 2020, Australia [13]	Multicenter, open-label RCT (May 2018 to July 2019)	216	Patients with sepsis-3 definition of septic shock in ICU	Intervention group: n = 109; IV vitamin C (1.5 g q6 hr); hydrocortisone (50 mg q6 hr); thiamine (200 mg q12 hr). Control group: n = 107; IV hydrocortisone (50 mg q6 hr) alone until shock resolution or up to 10 days	Primary outcome: duration of time alive and free of vasopressor administration up to day 7. Secondary outcomes: 28-day, 90-day, ICU, and hospital mortality, 28-day cumulative vasopressor-free days, 28-day cumulative mechanical ventilation-free days, 28-day renal replacement therapy-free days, change in SOFA score at day 3, 28-day ICU-free days, and hospital LOS
Iglesias et al., 2020, USA [14]	Single-Center RCT (February 2018 to June 2019)	137	Septic and septic shock patients admitted to ICU	Treatment group: n = 68; ascorbic acid IV 1500 mg q6 hr, thiamine 200 mg q12 hr, and hydrocortisone 50 mg q6 hr. Matching placebo: n = 69 for maximum of 4 days	Primary outcomes: resolution of shock, change in SOFA score. Secondary outcomes: 28-day mortality, ICU mortality, hospital mortality, procalcitonin clearance, hospital LOS, ICU LOS, ventilator-free days
Chang et al., 2020, China [15]	Single-center, single-blind RCT (September 2017 to January 2019)	80	Patients with sepsis or septic shock	Treatment group: n = 40; IV hydrocortisone (50 mg q6 hr for 7 days), vitamin C (1.5 g q6 hr for 4 days), and thiamine (200 mg q12 hr for 4 days). Placebo group: n = 40; normal saline	Primary outcome: 28-day all-cause mortality. Secondary outcome: organ protection, procalcitonin reduction, adverse events related to hydrocortisone, vitamin C, and thiamine
Moskowitz et al., 2020, USA [16]	Multicenter RCT (February 2018 to October 2019)	200	Adults aged ≥ 18 years with a suspected or confirmed infection and were receiving a vasopressor due to sepsis	Treatment group: n = 103; IV ascorbic acid 1500 mg, hydrocortisone 50 mg, and thiamine 100 mg q6 hr for 4 days. Placebo group: n = 102	Primary outcome: change in SOFA score between enrollment and 72 hr. Secondary outcome: renal failure, 30-day mortality
Wani et al., 2020, India [17]	Single-center, prospective, open-label RCT (April 2018 to June 2019)	100	Patients admitted with a diagnosis of sepsis and septic shock with a serum lactate level of >2 mmol/L	Treatment group: n = 50; IV vitamin C 1.5 g q6 hr for 4 days or until hospital discharge, hydrocortisone 50 mg q6 hr for 7 days or until ICU discharge followed by a taper over 3 days, and thiamine 200 mg q12 hr for 4 days or until hospital discharge. Control group: n = 50	Primary outcome: hospital mortality. Secondary outcome: 30-day mortality, duration of hospital stay, duration of vasopressor therapy, lactate clearance, change in serum lactate and SOFA score over first 4 days
Hwang et al., 2020, South Korea [18]	Multicenter RCT (December 2018 to January 2020)	111	Adult patients hospitalized and diagnosed with septic shock	Randomly assigned 1:1 to treatment or placebo group. Treatment group: n = 53; IV vitamin C 50 mg/kg (max single dose 3 g); thiamine 200 mg q12 hr for a total of 48 hr. Placebo group: n = 58; identical volume of 0.9% saline	Primary outcome: difference in SOFA score between enrollment and after 72 hr. Secondary outcome: shock reversal and 28-day mortality
Zabet et al., 2016, Iran [19]	Single-center RCT (September 2014 to January 2016)	28	Patients with septic shock who required vasopressors	Patients received 25 mg/kg IV ascorbic acid q6 hr; n = 14. Placebo group: n = 14. ascorbic acid IV: 25 mg/kg q6 hr for 72 hr	Primary endpoints: vasopressor dose and duration. Secondary endpoints: 28-day mortality, ICU LOS
Tanaka et al., 2000, Japan [20]	Single-center RCT (December 1992 to December 1997)	37	Patients with burns over >30% of total body surface area (TBSA) hospitalized within 2 hr of injury	Ascorbic acid group (n = 19; mean burn size 63% ± 26% TBSA; mean burn index 57 ± 26; inhalation injury 15/19). Control group (n = 18; mean burn size 53% ± 17% TBSA; mean burn index 47 ± 13; inhalation injury 12/18). Ascorbic acid IV: 25 mg/mL in RL initially in 24 hr, plus 66 mg/kg/hr based on TBSA burned and urine output	Primary endpoints: 24-hr total fluid infusion volumes, body weight, wound edema, severity of respiratory dysfunction. Secondary endpoints: hospital stay, length of mechanical ventilation, day of first surgery, no. of surgeries, no. of fasciotomies, no. of patients with pneumonia, mortality
Nabil and Ahmed, 2017, Egypt [21]	Prospective RCT	100	Adult patients admitted to the critical care department with the diagnosis of septic shock	Treatment group: n = 50; IV vitamin C 1.5 g q6 hr in the first 24 hr after admission until ICU discharge plus conventional sepsis treatment. Control group: n = 50, conventional sepsis treatment only	Primary outcome: vasopressor use, mechanical ventilation, renal replacement therapy, ICU LOS. Secondary outcome: ICU mortality
Nathens et al., 2002, USA [22]	Single-center RCT (February 1999 to June 2000)	595	91% of patients were victims of trauma, 16-74 years old, admitted to ICU under general surgery/trauma service	Patients randomized 1:1 to antioxidant supplementation (tocopherol and ascorbate) or routine care. Ascorbic acid IV: 1000 mg in 100 ml 5% dextrose q8 hr	Primary outcome: relative risk of pulmonary morbidity. Secondary outcomes: organ failure, duration of mechanical ventilation, ICU LOS
Marik et al., 2017, USA [23]	Single-center, retrospective before-after (January 2016 to July 2016)	47	Septic patients in ICU	Treatment group received IV ascorbic acid, thiamine, and hydrocortisone. Placebo control group. Ascorbic acid IV: 6 g/day in four doses or 1.5 g q6 hr	Primary endpoints: hospital mortality. Secondary endpoints: SOFA scoring, vasopressor duration, requirement of RRT, change in serum procalcitonin clearance, ICU LOS
Litwak et al., 2019, USA [24]	Retrospective cohort study (October 2016 to June 2018)	94	Adult patients with ICD 10 code for “septic shock” admitted to medical, surgical, or neurocritical care ICUs	Treatment group: n = 47; IV vitamin C 1.5 g q6 hr, hydrocortisone 50 mg q6 hr or 100 mg q8 hr, thiamine 200 mg q12 hr. Control group: n = 47, standard therapy and hydrocortisone	Primary outcome: hospital mortality. Secondary outcome: ICU mortality, ICU and hospital LOS, vasopressor duration, requirement for RRT, changes in serum creatinine, procalcitonin, lactate, and SOFA scores within the first 72 hr of initiation of vasopressor therapy
Sadaka et al., 2020, USA [25]	Multi-institutional, retrospective cohort study (March 2017 to September 2018)	62	Adults with a diagnosis of septic shock	Treatment group: n = 31; IV ascorbic acid 1.5 g q6 hr for 4 days, hydrocortisone 50 mg q6 hr for 7 days, and thiamine 200 mg q12 hr for 4 days. Control group: n = 31	Primary outcome: ICU and hospital mortality. Secondary outcome: ICU LOS, hospital LOS, RRT, vasopressor duration, mechanical ventilation-free days
Ahn et al., 2019, South Korea [26]	Retrospective cohort study (January 2017 to July 2017)	75	All patients admitted to the medical ICU for severe sepsis or septic shock and required mechanical ventilation within 24 hr from ICU admission	Treatment group: n = 35; IV vitamin C 2 g in 50 mL of 5% dextrose or normal saline q8 hr until ICU discharge. Control group: n = 40	Primary outcome: hospital mortality. Secondary outcome: ICU mortality, 90-day mortality, time to shock reversal, dose of vasopressors in first 4 ICU days, duration of initial mechanical ventilation, changes in SOFA scores and PaO_2_/FiO_2_ ratio in first 4 ICU days, use of RRT, ICU and hospital LOS
Mitchell et al., 2020, USA [27]	Retrospective cohort Study (March 2017 to July 2018)	76	Adult patients admitted to the medical or surgical ICU who received IV vitamin C, thiamine, and hydrocortisone	Treatment group: n = 38; IV vitamin C 1.5 g q6 hr and thiamine 200 mg q12 hr for 4 days, and hydrocortisone 50 mg q6 hr, 100 mg q8 hr, or continuous infusion of 10 mg/hr. Control group: n = 38 IV hydrocortisone alone	Primary outcome: hospital mortality. Secondary outcome: ICU, 28-day and 60-day mortality, ICU and hospital LOS, vasopressor duration, change in SOFA score over time
Park et al., 2020, Korea [28]	Single-center, retrospective study (January 2017 to July 2018)	178	Adults ≥ 18 years diagnosed with septic shock during ED stay and admitted to the ICU from the ED	Treatment group: n = 89; IV vitamin C 3 g q12 hr or 1.5 g q6 hr and thiamine 200 mg q12 hr in 50- or 100-mL of 5% dextrose or normal saline administered within 6 hr of shock recognition. Control group: n = 89, standard treatment	Primary outcome: delirium-free days. Secondary outcome: delirium incidence within 14 days, delirium coma-free days, delirium duration, hospital and ICU LOS, 28-day mortality
Shin et al, 2019, Korea [29]	Before and after study (October 2015 to December 2017)	1144	Septic shock registry with all adult patients (age ≥ 19 years) with septic shock who were diagnosed in an ED. October 2015 to June 2017 control group; July to December 2017 treatment group	Treatment group: n = 229; IV vitamin C 3 g q12 hr or 1.5 g q6 hr and thiamine 200 mg q12 hr in 50- or 100-mL of 5% dextrose or normal saline administered within 6 hr of shock recognition for 1 day. Control group: n = 915	Primary outcome: 28-day mortality. Secondary outcome: in-hospital mortality, hospital LOS, ICU LOS, duration of mechanical ventilation, and need for renal replacement therapy

**Figure 2:**
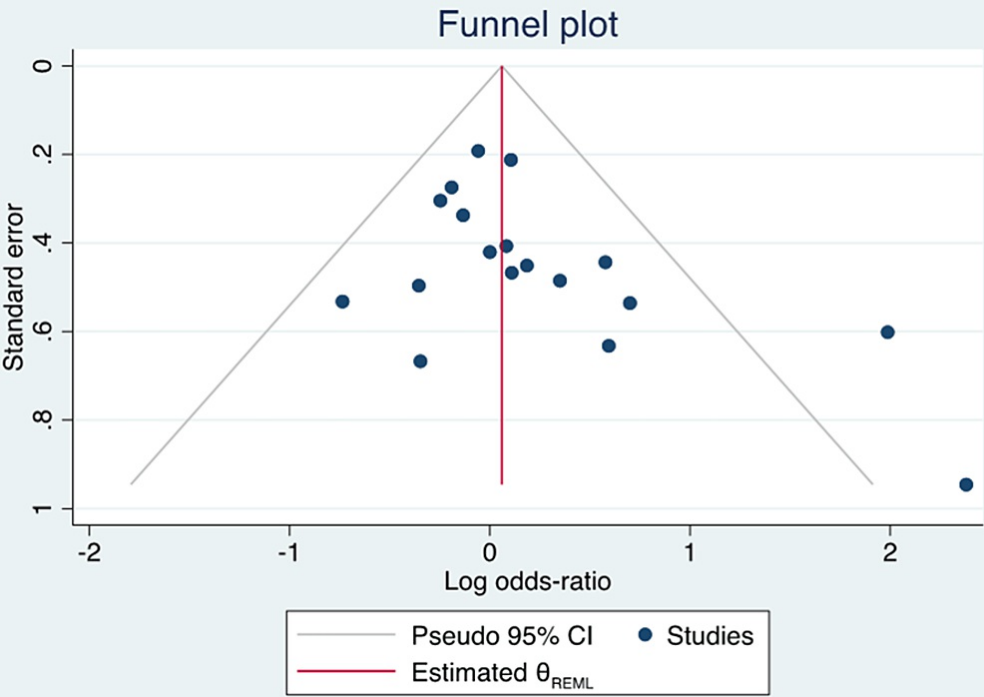
Funnel Plot Analysis.

Patient characteristics were relatively homogenous, with patients hospitalized for sepsis and/or septic shock. Six RCTs compared vitamin C, thiamine, and hydrocortisone to placebo [12-17]. Intervention arms of other RCTs included vitamin C with thiamine, IV vitamin C alone, and vitamin C with vitamin E [18-22]. Triple therapy (vitamin C, thiamine, and hydrocortisone) was the most common treatment regimen among retrospective cohort studies [23-25]. Funnel plot inspection demonstrated asymmetry, suggesting publication bias (Figure 2). In addition, the Harbord test was positive for publication bias (p = 0.04).

**Figure 3:**
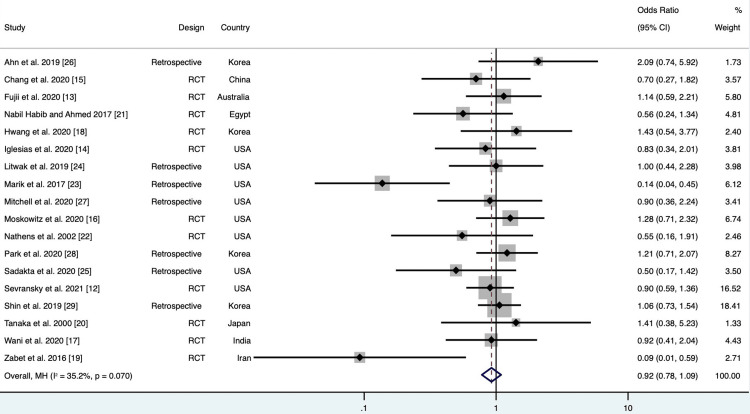
Forest plot demonstrating pooled mortality for vitamin C compared to standard of care. Note: Weights are from Mantel-Haenszel model

**Figure 4:**
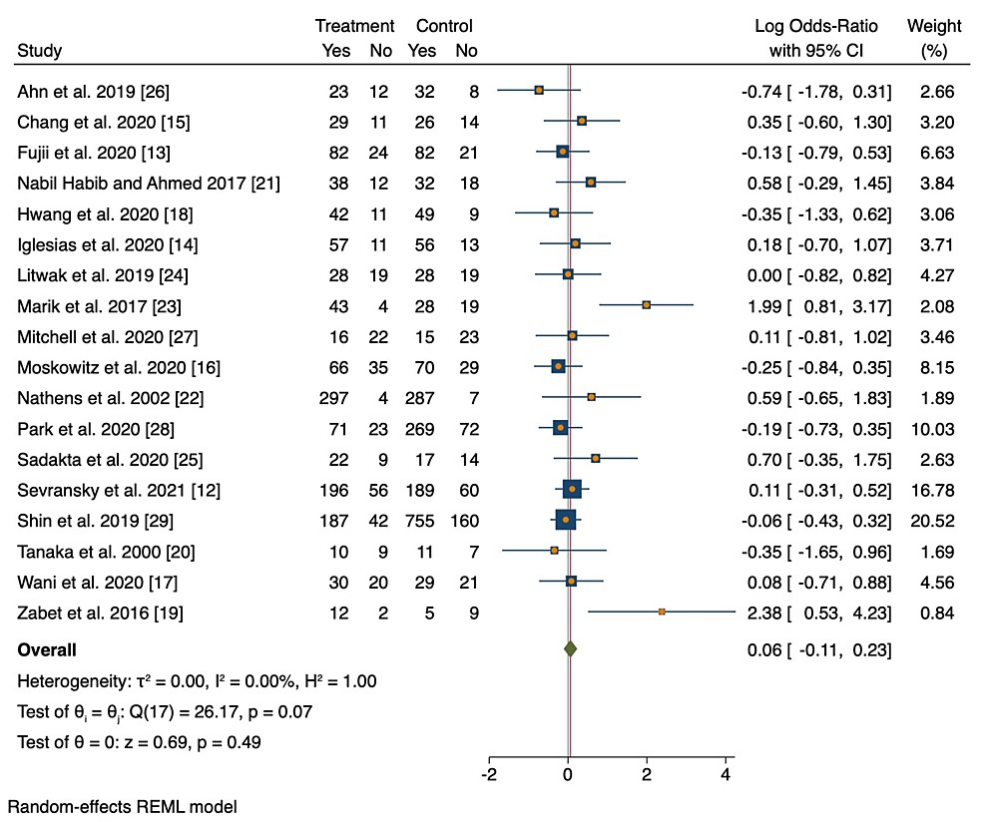
Random effects meta-analysis for mortality results.

**Figure 5:**
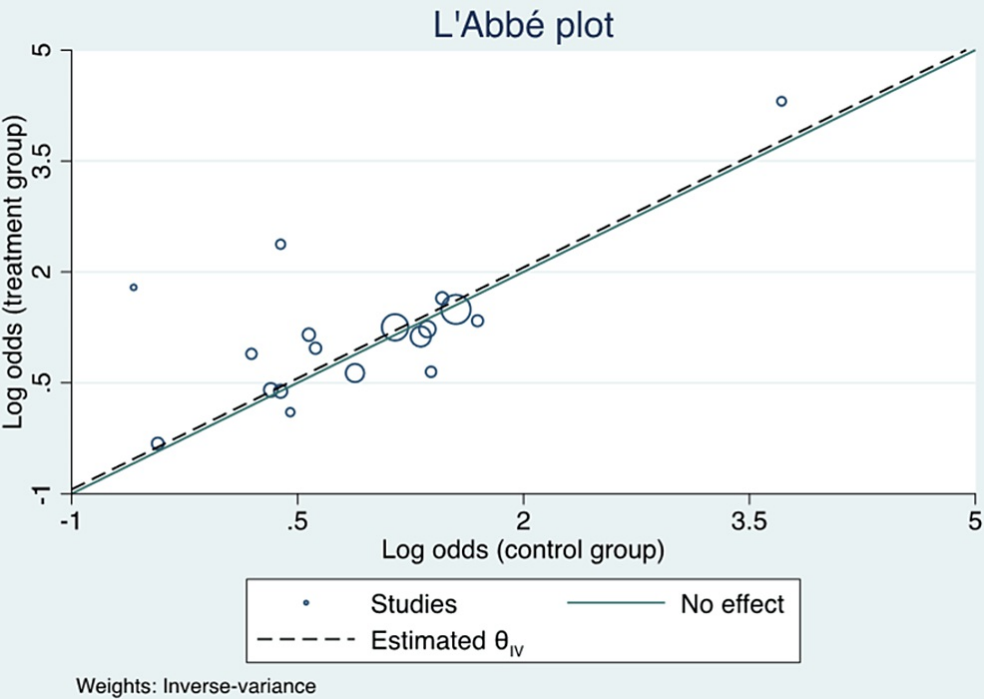
L'Abbé Plot.

**Figure 6:**
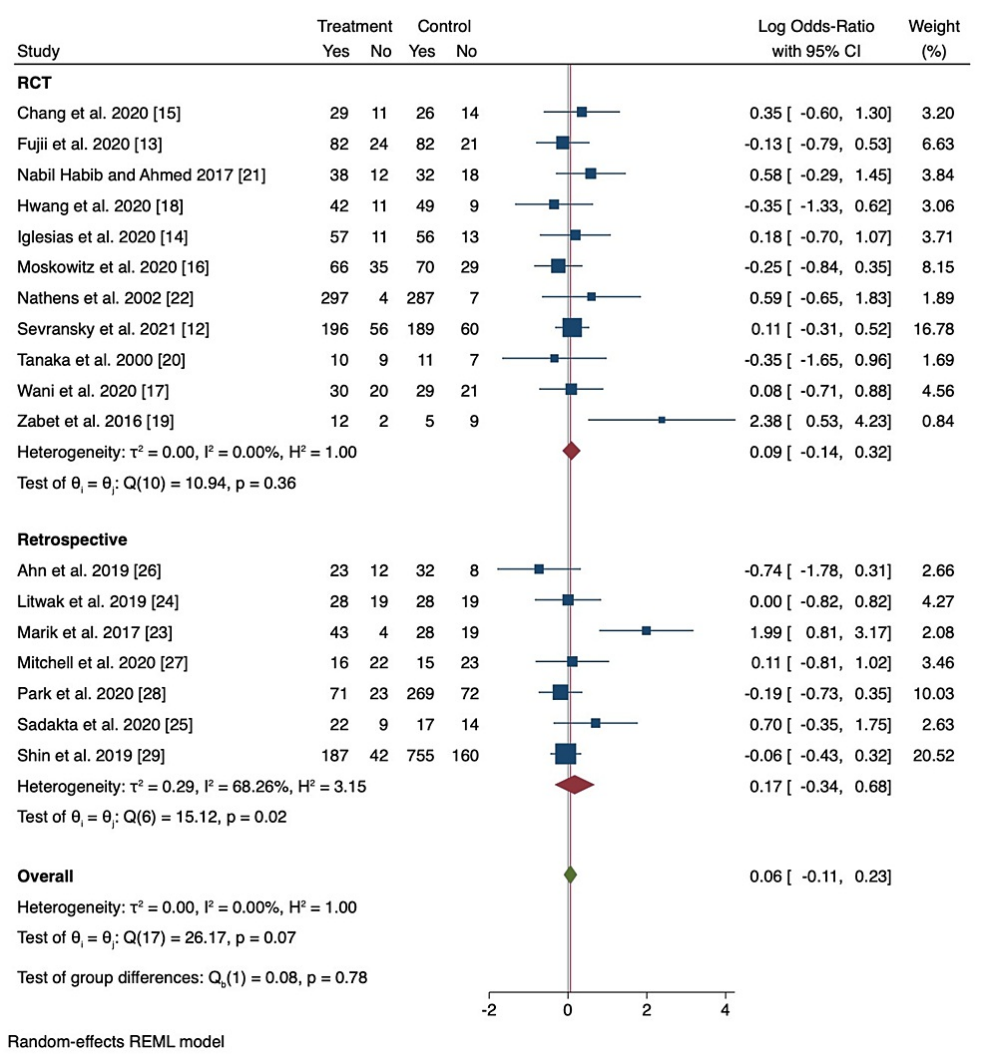
Pooled mortality subgroup analysis on study design.

**Figure 7:**
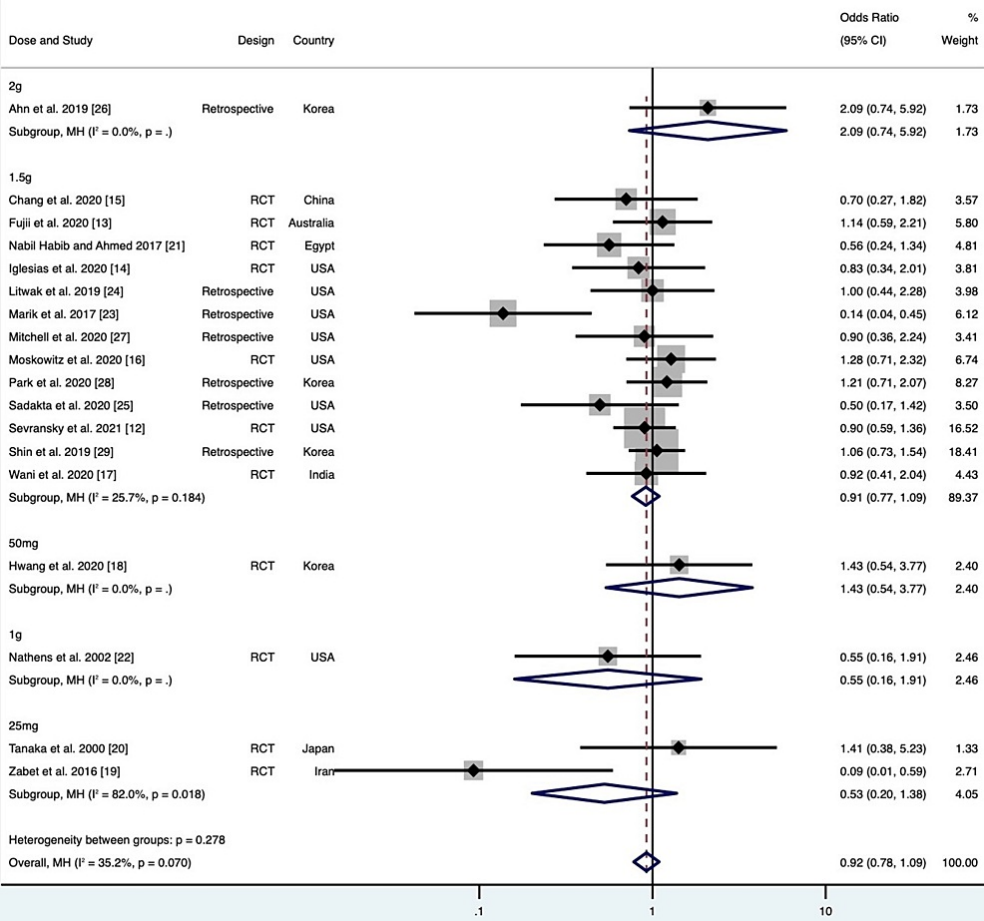
Pooled mortality subgroup analysis on vitamin C doses. Note: Weights and between-subgroup heterogeneity test are from Mantel-Haenszel model

Of the 18 studies included, pooled analyses did not show a statistically significant difference in mortality among patients treated with vitamin C compared to the standard treatment group (OR 0.92; 95% CI 0.78 to 1.09; p = 0.34; Figure 3). Results were the same after normalizing the distribution of subjects (log OR 0.06; 95% CI -0.11 to 0.23; p = 0.49; Figure 4). There was no evidence of major heterogeneity between all studies (I2 = 35.2%). On the L'Abbé plot, the majority of studies were around the line of no effect, confirming a lack of heterogeneity (Figure 5). There was moderate heterogeneity among retrospective studies (I2 = 60.5%). All studies were relatively evenly weighted, with the exception of the Sevransky 2021 study (% weight 15.62) and the Shin 2019 study (% weight 16.58) [12,29]. When comparing RCTs to retrospective studies, there was no difference in mortality between the two study designs (Figure 6). While 1.5 g every 6 hr of vitamin C was the most common dose among the included studies, there was no significant difference in mortality among different dosage groups (Figure 7).

Marik’s study found a significant difference in mortality among patients who received vitamin C, thiamine, and hydrocortisone (p < 0.001) with a propensity-adjusted odds of mortality in the treatment group being 0.13 [23]. In Zabet’s study, 28-day mortality was significantly lower in the vitamin C group compared to the control group (p = 0.009) [19]. None of the other studies found a statistically significant difference in mortality between the treatment and control groups. Fourteen studies reported ICU LOS with no significant difference (MD -0.77; 95% CI -4.67 to 3.12; p = 0.69). Eight studies reported the duration of mechanical ventilation, 13 studies reported SOFA score, and nine studies reported duration on vasopressors. There was no significant difference between both groups in regards to SOFA score (MD 0.38; 95% CI -1.98 to 2.73; p = 0.74, ), duration of mechanical ventilation (MD -1.75; 95% CI -9.99 to 6.48; p = 0.65), or duration on vasopressors (MD -0.89; 95% CI -2.23 to 0.46; p = 0.18).

Discussion

Our meta-analysis examined the proposed antioxidant effects of vitamin C as adjuvant therapy for sepsis and septic shock. Parenteral vitamin C alone or as adjuvant therapy with thiamine and hydrocortisone did not show a reduction in 28-day mortality, ICU LOS, duration of mechanical ventilation, duration of vasopressors, or change in SOFA score. Our results were consistent with recent RCTs showing no mortality benefit of combination therapy with vitamin C, thiamine, and hydrocortisone [12-18]. Contrary to our findings, Zabet’s randomized, double-blind, placebo-controlled clinical trial showed a decreased 28-day mortality in the ascorbic acid group [19]. This study had important limitations including a small sample size of 28 patients, of which 14 patients received vitamin C and lack of a baseline ascorbic acid level. Marik et al.’s retrospective before-after clinical study found a mortality benefit although the study enrolled both patients with sepsis and septic shock. This suggested that the study enrolled a less critically ill population as evidenced by a lower mean baseline SOFA score (8.3 ± 2.8) compared to randomized clinical trials such as the VICTOR trial where the mean SOFA score was significantly higher (baseline SOFA 11.22 ± 2.99) and showed no improvement in mortality with hydrocortisone, vitamin C, and thiamine [23].

The most common dosing interval was 1.5 g of vitamin C every 6 hr. Treatment regimens did not make a difference, as evident in Ahn et al.’s study where 2 g of vitamin C every 8 hr did not demonstrate a mortality benefit [26]. Since our study did not show signs of overall heterogeneity (although moderate heterogeneity was found among retrospective studies), we believe most of the studies included for analysis were consistent.

Patients in septic shock present with severe hypovitaminosis C which was evident throughout multiple studies which required high-dose vitamin C infusions in order to correct plasma concentrations. However, it is unknown whether septic patients with a benign clinical presentation would benefit from supraphysiologic doses. Further studies with a larger patient population are needed to provide stronger evidence for use of vitamin C and determine which subgroups of patients would benefit from parenteral administration.

Our meta-analysis was comprehensive, with the inclusion of secondary outcomes to determine if vitamin C had an impact on multiple clinical endpoints during hospitalization. This is in contrast to previous meta-analyses which focused primarily on mortality. In addition, we performed various subgroup analyses of study design (observational studies versus RCTs) and dose intervals. Our results were consistent with a prior meta-analysis that found no reduction in pooled mortality with vitamin C administration [30]. In addition, a recent meta-analysis of RCTs that evaluated combination therapy with hydrocortisone, vitamin C, and thiamine found no association with reduction in mortality or ICU LOS although it showed a reduction in SOFA score on day 3 post-randomization [31].

Limitations

Several limitations were noted in our meta-analysis. Subgroup analyses based on illness severity and comorbidities were not performed. Studies did not categorize patients based on levels of sepsis, thus it was not possible to conduct a subgroup analysis on patients with sepsis versus septic shock. 11 studies only selected patients admitted to the ICU for septic shock. Some clinical trials lacked generalizability due to the inclusion of a specific, homogenous population such as critically ill surgical or trauma patients [20,22]. In addition, the Vitamin C, Thiamine and Steroids in Sepsis (VICTAS) RCT only included patients with sepsis-induced cardiovascular or respiratory failure [12]. We included studies with performance and detection bias with a lack of placebo and blinding among investigators, leading to the potential for bias when evaluating endpoints. Also, several trials allowed for corticosteroids to be administered at the clinician’s discretion in the control group, as seen in 33% in the VICTAS trial, 41% in the ORANGES (outcomes of metabolic resuscitation using ascorbic acid, thiamine, and glucocorticoids in the early treatment of sepsis) trial, and 100% in the VITAMINS (vitamin C, Hydrocortisone and Thiamine in Patients With Septic Shock) trial [12-14]. Furthermore, combination treatment regimens were heterogeneous which may have impacted results. More recent RCTs included hydrocortisone, ascorbic acid, and thiamine therapy due to the proposed synergistic effect on the immune system and mitochondrial oxidative function [23]. Of note, a few RCTs were slightly underpowered due to early termination [12,15].

## Conclusions

In conclusion, we assessed recently published RCTs and retrospective studies investigating the use of vitamin C in treating sepsis. We determined that there was a lack of evidence to support the effectiveness of vitamin C in treating patients with sepsis. Further research is required to investigate the value of supraphysiologic doses of parenteral vitamin C before it can be recommended for use in the management of sepsis.
